# Roles of 5,10-Methylenetetrahydrofolate Reductase C677T Polymorphisms in First-Episode, Drug-Naive Adult Patients With Depression

**DOI:** 10.3389/fpsyt.2020.531959

**Published:** 2020-12-07

**Authors:** Zhuoqing Li, Bo He, Jian Xu, Nan Dai, Liangliang Ping, Cong Zhou, Zonglin Shen, Xiufeng Xu, Yuqi Cheng

**Affiliations:** ^1^Department of Psychiatry, First Affiliated Hospital of Kunming Medical University, Kunming, China; ^2^Department of Medical Imaging, First Affiliated Hospital of Kunming Medical University, Kunming, China; ^3^Department of Internal Medicine, First Affiliated Hospital of Kunming Medical University, Kunming, China; ^4^Department of Psychiatry, Xianyue Hospital, Xiamen, China

**Keywords:** major depressive disorder, MTHFR C677T, cortical thickness, subcortical structure volume, anterior cingulate cortex

## Abstract

5,10-Methylenetetrahydrofolate reductase (MTHFR) gene C677T polymorphism is considered as a predisposition and promising genetic candidate to major depressive disorder (MDD), as it is associated with impaired one-carbon cycles, which may be involved in the pathogenesis of depression. Cortical thickness (CT) and subcortical structure volumes have been extensively studied in MDD and have been proposed as one of the phenotypes for MDD. We intend to discuss the association between CT, subcortical structure volume, and *MTHFR* C677T polymorphism in first-episode, treatment-naive patients with MDD. In this study, 127 adult patients with MDD and 101 age- and gender-matched healthy controls (HCs) were included. All subjects underwent T1-weighted MRI, *MTHFR* C677T genotyping, and FreeSurfer software-based morphological analysis. MDD patients have been detected to have significantly decreased volumes in the left nucleus accumbens (*P* < 0.001). The *MTHFR* 677 T allele carriers manifested with thinner CT in the left caudal anterior cingulate cortex (cACC, *P* = 0.009) compared with CC genotype. There were significant genotype-by-diagnosis interactions for the CT in the left cACC (*P* = 0.009), isthmus cingulate (*P* = 0.002), medial orbitofrontal lobe (*P* = 0.012), posterior cingulate (*P* = 0.030), and the right lateral orbitofrontal lobe (*P* = 0.012). We also found a trend in the interaction effect on the volume of the left putamen (*P* = 0.050). Our results revealed that *MTHFR* C677T polymorphism may be involved in the dysfunction of limbic–cortical–striatal–pallidal–thalamic (LCSPT) circuits mediating emotion processing, which may contribute to pathogenesis of MDD.

## Introduction

Major depressive disorder (MDD) is a chronic persistent complex mental disorder with high morbidity. The etiology and pathogenesis of MDD are still unclear. Current evidence has indicated that MDD is mainly the outcome of the genetic and environment interactions, such as adverse experiences in childhood, especially childhood maltreatment (1). Family, adoption, and monozygotic- and dizygotic-twin studies have supported that both genetic and environmental factors and their interconnection contribute to MDD, and the heritability of MDD was about 37% (2, 3) Previous multiple meta-analyses have shown that 5,10-methylenetetrahydrofolate reductase (*MTHFR*) C677T confers a predisposition to depression (4–7).

The MTHFR enzyme mainly expresses in the brain and fetal and frontal cortex, and it is an irreversible rate-limiting enzyme in the essential methyl cycle of the body. MTHFR critically catalyzes the conversion of 5, 10-methylenetetrahydrofolate to 5-methyltetrahydrofolate, a co-substrate for homocysteine (Hcy) remethylation to methionine, which then is activated as *S*-adenosyl methionine (SAM). *MTHFR* C677T has a prominent function in one-carbon metabolism (1-CM); the consequences of perturbed 1-CM include reduced remethylation of total Hcy (tHcy) into methionine, elevated Hcy levels, and a reduction in cellular methylation potential (8). Research on *MTHFR* C677T (9) has shown that MTHFR activity decreased in a T allele dose-dependent form. Defects in the MTHFR enzyme can cause methionine synthesis obstacles for Hcy. The synthesis of SAM from methionine in the organism decreases, and the methylation reaction decreases. High Hcy levels, through triggering the *N*-methyl-d-aspartate receptor (10), lead to monoamine neurotransmitter synthesis being blocked and essential amino acid levels being reduced in the brain tissue, especially neurotransmitters implicated in depression (such as serotonin, norepinephrine, glutamate, and γ-aminobutyric acid).

A meta-analysis substantiated that *MTHFR* C677T had the most prominent risk effect of depression in Asians (11); this effect is more prominent in the Chinese population (12). However, the results were inconsistent with some previous studies. For example, a meta-analysis study including only European Caucasian populations did not find that *MTHFR* C677T polymorphism was associated with recurrent depression (13), and there was a study suggesting that the C677T genotype may be a protector for MDD (14), yet most evidence from meta-analyses strongly suggested that the T allele or TT genotype tends to be a risk effect to MDD (5, 15). The inconsistent results may be due to clinical heterogeneity, ethnicity, geography, age, size of the sample, medication confounding factors, lack of statistical efficiencies, and/or the interaction of *MTHFR* C677T with other genes. Gene × gene studies deduced that by affecting the methyl donor SAM, *MTHFR* C677T exhibited a coordinated effect with some genes which may be involved in the monoamine neurotransmitter, dopaminergic pathway, and regulation of neuroplasticity, via diminished methyl (15). For instance, 5-HTT can remove 5-HT from the synaptic cleft, which in turn determines the amount and duration of postsynaptic membrane receptor-mediated signaling. *5-HTT* is often a target gene for epigenetic modification methylation, regulating the relationship between stress and depression (16, 17). *MTHFR* C677T can cause a decrease in the methyl level of the CpG island of the SLC6A4 promoter, increasing the expression and promoting the increase of 5-HTT activity and thus the reduction of synaptic 5-HT. Similarly, *MTHFR* C677T boosts the expression of the *COMT* gene and enhances the action of methylase to block the dopaminergic signaling pathway (18). Besides, the *MTHFR* C677T allele attenuates the degradation of norepinephrine and dopamine induced by high-efficiency methylases such as the COMT Val risk gene. The interaction between the *MTHFR* C677T and *COMT* gene determines the final effect of the genes, which will significantly increase the risk of depression (19) and correlate with the decrease in the volume of the putamen of elderly depression (20). On the other hand, gene × environmental studies have found that *MTHFR* C677T downregulates the expression of the *Sirtuin1* gene (*SIRT1*) with axonal growth, neurogenesis, and dendritic branching via high Hcy, hence accelerating endothelial progenitor cell aging (21). The level of DNA methyl in the promoter region of the glucocorticoid receptor gene (*NR3C1*) was observed to be reduced in MDD patients (22). Under stress, the DNA methylation of specific alleles in the glucocorticoid responsive element of the FKBP5 gene polymorphism was reduced, resulting in increased expression of the corresponding gene, glucocorticoid receptor inactivation, and glucocorticoid resistance (23). In short, *MTHFR* C677T may participate in the development of depression through epigenetic DNA methylation modifications, metabolic disorders, and neurotransmitter disturbances together with some genes and/or environments (such as stress). In addition, the MTHFR defect caused by *MTHFR* C677T can be restored to normal by available supplements (24), which is clinically significant.

Genetic imaging may be an advanced method to screen for reliable and heritable biomarkers in neuropsychiatric disorders (25). Previous studies have found that *MTHFR* C677T polymorphism with high Hcy level was associated with decreased cortical thickness (CT), subcortical gray matter (GM), and white matter volume and/or density in patients with mental illness (18, 20, 26, 27). The refinement/efficiency of the connections between prefrontal and subcortical structures could alter the emotion processing and regulation (28). Thus, we speculated that *MTHFR* C677T may affect brain connections and be associated with depression. However, little is known about the role of *MTHFR* C677T polymorphism in the MDD.

In this study, we intend to explore the potential role of *MTHFR* C677T polymorphism on the brain in first-onset adult depression, mainly in terms of the structure of brain gray matter. We used the FreeSurfer brain imaging technology to measure cerebral cortex thickness and subcortical gray matter volume to evaluate changes in brain gray matter structure.

## Materials and Methods

### Participants

Two experienced attending psychiatrists selected the patients with depression that met the inclusion criteria from the psychiatric clinic of the First Affiliated Hospital of Kunming Medical University. We collected some basic information about all subjects, such as age, gender, education level, ethnicity, dominant hand, and patients' course of disease. The inclusion criteria were as follows: (1) meeting *Diagnostic and Statistical Manual of Mental Disorders*, fourth edition (DSM-IV) (29), diagnostic criteria for MDD, using the Structured Clinical Interview for DSM-IV Axis I disorders (SCID-1) (30) to improve its validity; (2) adults aged 18–45 years; (3) first-onset and untreated depression (without drug, physical, and standard psychological treatment); (4) a total score of ≥17 in the 17-item Hamilton Depression Rating Scale (HAMD-17) (31); and (5) being a Chinese Han, right handed, and a Kunming native. The exclusion criteria were as follows: (1) suffering from mental illnesses other than depression; (2) history of alcoholism, traumatic brain injury, or severe physical diseases; (3) and any contraindication for MRI. The healthy control (HC) participants were generally healthy, were aged 18–45 years, were recruited from local communities with SCID-1, and were well-matched to the patient group on gender, age, ethnicity, dextromanuality, and birthplace. All subjects underwent HAMD-17 (31), Hamilton Anxiety Scale (32) (HAMA) evaluation, *MTHFR* C677T genotyping, and T1-weighted MRI scans. Data of an abnormal brain structure visible to the naked eye or poor-quality images affected by head movement were eliminated. For detailed data, refer to Table 1. The study was approved by the Institutional Review Board of Kunming Medical University, Yunnan Province, China. All participants gave written informed consent.

**Table 1:** Demographics and clinical characteristics of patients with MDD and HC.

	**MDD**	**HC**	**χ^**2**^/*t***	***P*-Value**
	**(*n* = 127)**	**(*n* = 101)**		
Gender (male/female)	43/84	45/56	2.716	0.099
Age (year)	31.39 ± 7.87	29.83 ± 5.84	1.711	0.088
Years of education	12.22 ± 4.16	16.15 ± 4.32	−6.964	<0.001
eTIV (mm^3^)	1188703.41 ± 389654.26	1331694.02 ± 181989.74	−3.663	<0.001
HAMD score	23.81 ± 5.01	0.39 ± 0.63	52.165	<0.001
HAMA score	22.96 ± 6.01	0.66 ± 0.77	41.506	<0.001
Illness duration (months)	12.31 ± 16.60			
***MTHFR*** **gene polymorphism C677T**				
CC	39	40	3.663	0.160
CT	69	42		
TT	19	19		
HWE for MDD			1.658	0.198
HWE for HC			1.726	0.189
CC	39	40	1.966	0.161
CT+TT	88	61		
C(allele frequency)	147	122	0.296	0.587
T(allele frequency)	107	80		

*MDD, major depressive disorder; HC, healthy control; eTIV, Estimated Total Intracranial Volume; HAMD, Hamilton Depression-17 item scale; HAMA, Hamilton Anxiety Scale; HWE, Hardy-Weinberg equilibrium test; CC, CC genotype of C677T; TT + TC, TT or TC genotype of C677T*.

*Data are means ± standard deviation for age, years of education, eTIV, HAMD, and HAMA scores, and duration of illness*.

*The P-values for distributions of gender, MTHFR C677T genotype, C/T allele frequency, were obtained by chi-square test. The P-values for comparisons of age, years of education, eTIV, HAMD, and HAMA scores, and duration of illness were obtained using independent t-tests. Allele frequencies (C/T allele): MDD patients 0.58/0.42, HC subjects 0.60/0.40*.

### Measurements

Treatment guidelines for depression suggest that it is important to consider severity when selecting a patient's initial treatment modality. HAMD has been the most frequently used scale to subdivide patients into severity groups and examine the treatment implications of symptom severity (31). HAMA is often used to assess anxiety symptoms and severity (32). Patients with depression are often with or without anxiety symptoms, and the two often have comorbidities. In this study, we mainly study patients with depression with or without anxiety. Thus, two professional psychologists evaluated HAMD-17 and HAMA scales on all subjects. The cutoff score on the HAMD that maximized the sum of sensitivity and specificity was 17 for the comparison of mild vs. moderate depression and 24 for the comparison of moderate vs. severe depression (33).

### Genotyping

Genomic DNA was extracted from the subject's vein using the AxyPrep™ Blood Genomic DNA Miniprep Kit and stored at −80°C until use. All the extracted genes were sent to the Beijing Huada Biological Company for genotyping. Huada used the principle of MassARRAY technology, with its core matrix-assisted laser desorption ionization time-of-flight mass spectrometry (MALDI-TOF MS) technology, along with the corresponding Sequenom to detect the *MTHFR* C677T locus genotype. The genotyping completion rate in our study was 100%. Genotype frequencies of *MTHFR* C677T were used to calculate the Hardy–Weinberg equilibrium, applying the online software SHEsis (34). The details are described in Table 1.

### MRI Data Acquisition

MRI data were acquired at Philips Achieva 3.0-T TX. Restraining foam pads were used to minimize head motion. 3D T1-weighted MRI-prepared rapid gradient-echo (MP-RAGE) was used with the following parameters to obtain brain structure data: 7,380-ms repetition time, 3.4-ms echo time, 250 × 250-mm field of view, 256 × 256 matrix size, 1.2-mm slice thickness, 230 coronal slices without gap, 90° flip angle, and 6-min 53-s scan duration time. T1- and T2-weighted magnetic resonance images were checked first to exclude those with significant structural abnormalities.

### Image Processing

All primary DICOM images were converted into the NIfTI format using an MRI conversion software (http://lcni.uoregon.edu/downloads/mriconvert/mriconvert-and-mcverter). All the structural data were analyzed with the FreeSurfer 5.3 Development Version (Massachusetts General Hospital, Boston, http://surfer.nmr.mgh.harvard.edu). Cortical reconstructions were visually inspected to check for the accuracy of the automatic segmentation of the gray/white matter boundary, and no scans required manual editing. The average CT was defined as the shortest distance from the known apex of the cortex between the pial surface and the gray/white matter junction (35). A Gaussian kernel of 10-mm full width at half-maximum was applied to the subjects' CT maps. Each hemisphere was automatically parcellated into 34 distinct cortical regions according to the Desikan–Killiany atlas (36). The CT value was measured by calculating the distance between the white matter and pial surfaces at 160,000 vertices in each hemisphere in FreeSurfer. We used the automatic segmentation pipeline in FreeSurfer to calculate the subcortical volume data including volumes of the bilateral putamen, caudate nucleus, globus pallidus, nucleus accumbens (NAC), thalamus, amygdala, and hippocampus. We extracted CT values of the 34 regions and seven subcortical volumes' data in each hemisphere for subsequent statistical analysis.

### Statistical Analyses

In the main analysis, CT and volume values were compared between MDD, HC, and *MTHFR* C677T genotype groups (CC vs. TT+TC), and then the genotype-by-diagnosis interaction was further investigated. The genotype groups were based on the dominant model (comparing risk allele with the non-risk allele carriers) in accordance with previous imaging studies on the *MTHFR* gene (37). The general linear model (GLM), two-way analysis of covariance (ANCOVA), and full factorial model were performed in SPSS 17.0, evaluating the main effect of “diagnosis,” “genotype,” and the interactive effect of “diagnosis × genotype.” CT/subcortical volume values were selected as the dependent variables, diagnosis and genotype as the independent variables, and age, gender, years of education, and estimated total intracranial volume (eTIV) as covariates, according to the statistical procedure used in a previous similar study (38). In the primary analysis, the test level was set to α = 0.05, two-sided test. Regions with statistically significant differences in the above ANCOVA analysis were further analyzed in pairs. As an exploratory investigations study, according to a previous research (ref), we set up multiple correction levels according to previous imaging genetic studies (18). Therefore, the multiple comparisons' correction test level for this study was relaxed to α_diagnosis_ = 0.01, α_genotype_ = 0.05, and α_interaction_ = 0.05, two-sided test.

Secondly, we aimed to analyze the specific role of the C677T allele in brain structural changes of depression. Brain regions with statistically significant differences in the analysis of diagnosis × genotype interactions were chosen as regions of interest (ROIs), and the *post-hoc* tests for ROIs were performed later. With reference to a previous study (38), we compared the ROIs between MDD patients and the HC participants within each genotype group using a one-way ANCOVA, controlling for the same covariates as the main analysis.

The clinical features of depression in the MDD group (e.g., duration of disease, HAMD score, and HAMA score) were examined in relation to CT or subcortical volume values of ROIs. We conducted partial correlation analysis, controlling for age, gender, years of education, eTIV, and genotype. A height threshold of *P* < 0.05 was set.

The distributions of age, years of education, eTIV, HAMD score, and HAMA score between MDD and HC groups or *MTHFR* C677T genotype TT+CT vs. CC, as well as the duration of disease between genotype groups in patients with MDD, were analyzed using independent *t*-tests. The chi-square test was used to compare the gender and genotype distributions. The significance threshold was set to *P* < 0.05. Statistical analyses were performed using SPSS version 17.0 (SPSS Inc., Chicago, IL).

## Results

### Demographic and Genotypic Characteristics

We could not find any significant difference between the patients and the HCs in terms of age, gender, and genotype distribution except for years of education (*t* = −6.964, *P* < 0.001), HAMD (*t* = 52.165, *P* < 0.001), HAMA (*t* = 41.506, *P* < 0.001), and eTIV (*t* = −3.663, *P* < 0.001). Comparing years of education, eTIV, HAMD score, and HAMA score between genotype groups of TT+CT and CC, we could not find any significant difference, except for age (*t* = −2.384, *P* = 0.018) and gender (χ^2^ = 10.792, *P* = 0.001). Compared to C homozygote, the T carrier group appeared younger and had more males. There was no significant difference between the genotype of CC and T carriers within the MDD group with regard to illness duration, HAMD score, and HAMA score. Detailed information is presented in Tables 1–3.

**Table 2:** Demographics and clinical characteristics of the subjects with *MTHFR* T-carrier and C-homozygous.

	**TT+CT**	**CC**	**χ^**2**^/*t***	***P*-Value**
	**(*n* = 149)**	**(*n* = 79)**		
Gender (male/female)	69/80	19/60	10.792	0.001
Age (year)	29.89 ± 6.97	32.22 ± 7.06	−2.384	0.018
Years of education	14.07 ± 4.71	13.75 ± 4.56	0.504	0.615
Ethnic Han (%)	100%	100%		
Dextromanuality (%)	100%	100%		
eTIV (mm^3^)	1.2570E6 ± 3.4432E5	1.2426E6 ± 1.2570E6	0.318	0.751
HAMD score	14.107 ± 12.044	12.165 ± 12.614	1.140	0.255
HAMA score	13.738 ± 11.847	11.810 ± 12.1540	1.159	0.248

*eTIV, Estimated Total Intracranial Volume; HAMD, Hamilton Depression-17 item scale; HAMA, Hamilton Anxiety Scale; T-carrier, The TT genotype or CT genotype of MTHFR C677T polymorphism; C-homozygous, CC genotype of MTHFR C677T polymorphism*.

*Data are means ± standard deviation for age, years of education, eTIV, HAMD, and HAMA scores*.

*The P-value for distributions of gender was obtained by chi-square test. The P-values for comparisons of age, years of education, eTIV, HAMD, and HAMA scores, were obtained using independent t-tests*.

**Table 3:** The illness duration and disease severity assessment of different genotypes in MDD.

	**CT+TT (*n* = 88)**	**CC (*n* = 39)**	***t***	***P*-Value**
HAMD	23.58 ± 4.98	24.33 ± 5.11	0.781	0.436
HAMA	22.77 ± 6.05	23.40 ± 5.95	0.463	0.644
Illness duration (months)	12.74 ± 18.05	11.34 ± 12.89	0.438	0.662

*Data are means ± standard deviation for HAMD, HAMA scores, and illness duration*.

### Differences on CT and Volume of Subcortical Values in the Whole Brain According to Diagnosis and *MTHFR* C677T Genotype

The statistically significant results of the 2 × 2 ANCOVA and full factorial model between MDD and HC and between T carrier and C-homozygous groups on CT and volume values (CT values of the 68 regions and 14 subcortical volumes' data in whole brain) are described in Table 4. Patients with MDD showed significantly smaller volume of the subcortical NAC (*F* = 14.968, *P* < 0.001, which remained significant after Bonferroni correction) in the left hemisphere compared with the HC group. However, no significant difference in CT values (*P* > 0.01) between patients with MDD and the HC group was found. In the comparison of genotype groups, *MTHFR* C677T allele carriers exhibited significantly reduced CT in the left caudal anterior cingulate cortex (cACC, *F* = 6.933, *P* = 0.009), rostral anterior cingulate cortex (rACC, *F* = 4.090, *P* = 0.044), frontal pole (*F* = 4.099, *P* = 0.044), right cACC (*F* = 5.663, *P* = 0.018), and supramarginal gyrus (*F* = 4.425, *P* = 0.037) compared with the C-homozygous group. In contrast, *MTHFR* C677T allele carriers manifested significantly increased CT in the left cuneus (*F* = 6.646, *P* = 0.011) and pericalcarine (left, *F* = 6.565, *P* = 0.011; right, *F* = 4.961, *P* = 0.027) in hemispheres on both sides. T allele carriers and the C-homozygous group did not differ significantly in terms of subcortical volume (*P* > 0.05). Significant diagnosis-by-genotype interaction effects on CT presented in the cACC (*F* = 6.926, *P* = 0.009), isthmus cingulate (ISTC, *F* = 10.257, *P* = 0.002), medial orbitofrontal cortex (mOFC, *F* = 6.457, *P* = 0.012), and posterior cingulate cortex (PCC, *F* = 4.751, *P* = 0.030) in the left hemisphere and in the right lateral orbitofrontal cortex (lOFC, *F* = 6.452, *P* = 0.012).

**Table 4:** Statistically significant diagnostic main effect, genotype main effect, and diagnosis × genotype interaction effect in the respect of the whole cortical thickness and subcortical gray matter volume.

**Region**	**Diagnosis** **MDD (*****n*** **=** **127) vs. HC (*****n*** **=** **101)**			**Genotype** **CC (*****n*** **=** **79) vs. TT+CT (*****n*** **=** **149)**			**Diagnosis** **×** **Genotype**	
	***F***	***P***		***F***	***P***		***F***	***P***
**Cortical thickness (mm)**								
L caudal anterior cingulate	1.102	0.295		6.933	0.009**	CC>CT+TT	6.926	0.009**
L rostral anterior cingulate	3.225	0.074		4.090	0.044*	CC>CT+TT	2.611	0.108
L frontal pole	0.998	0.319		4.099	0.044*	CC>CT+TT	0.578	0.448
L cuneus	1.560	0.213		6.646	0.011*	CC < CT+TT	0.060	0.808
L pericalcarine	0.349	0.555		6.565	0.011*	CC < CT+TT	1.380	0.241
L isthmuscingulate	0.001	0.971		0.238	0.626		10.257	0.002**
L medial orbitofrontal	0.669	0.414		0.093	0.761		6.457	0.012*
L posterior cingulate	0.223	0.637		1.258	0.263		4.751	0.030*
L caudal middle frontal	4.181	0.042*	HC>MDD	1.195	0.276		0.025	0.875
R inferiorparietal	4.442	0.036*	HC>MDD	0.298	0.586		0.499	0.481
R inferiortemporal	5.343	0.022*	HC>MDD	1.649	0.200		0.005	0.945
R postcentral	4.057	0.045*	HC>MDD	0.082	0.774		0.027	0.869
R lateral orbitofrontal	5.818	0.017*	HC < MDD	0.010	0.920		6.452	0.012*
R caudal anterior cingulate	0.015	0.903		5.663	0.018*	CC>CT+TT	1.449	0.230
R supramarginal	1.342	0.248		4.425	0.037*	CC>CT+TT	3.094	0.080
R pericalcarine	0.919	0.339		4.961	0.027*	CC < CT+TT	0.011	0.916
**Volume of subcortical gray matter (mm**^**3**^**)**								
L putamen	1.080	0.300		0.016	0.900		3.872	0.050*
L amygdala	5.130	0.024*	MDD < HC	0.778	0.379		1.327	0.251
L nucleus accumbens	14.968	0.000***^a^	MDD < HC	0.532	0.466		3.169	0.076

*MDD, major depressive disorder; HC, healthy control; T-carrier, The TT genotype or CT genotype of MTHFR C677T polymorphism; C-homozygous, CC genotype of MTHFR C677T polymorphism; L, left hemisphere; R, right hemisphere*.

P < 0.05*, P < 0.01**, P < 0.001***.

*The F and P-values were obtained using two-way analysis of covariance adjusted for age, gender, years of education, and eTIV as covariates*.

*Bonferroni correction was applied in the analyses of diagnosis effect (MDD vs. HC), genotype effect (TT + CT vs. HC) and diagnosis-by-genotype interaction*.

a *Cortical regions that remained significant after Bonferroni correction are marked with an asterisk*.

**Figure 1:**
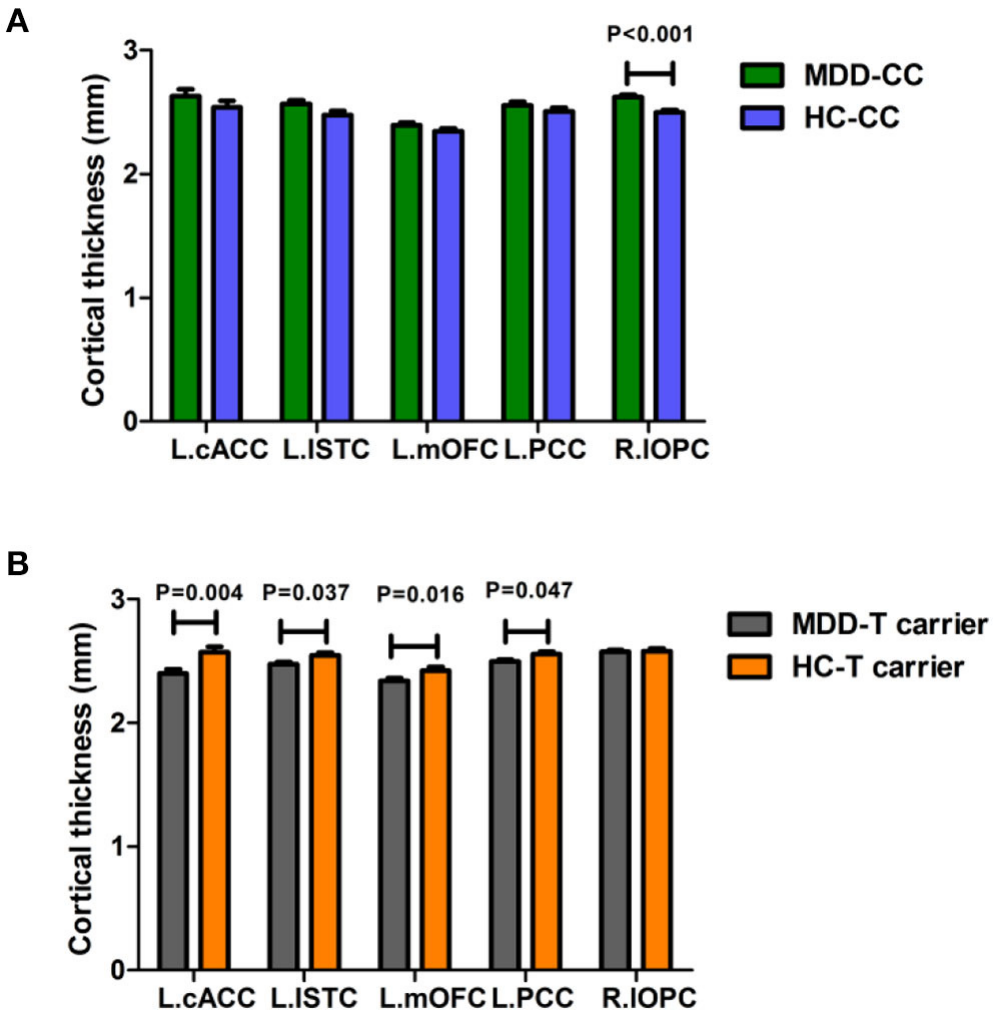
Comparison of the cortical thickness values of the left cACC, ISTC, mOFC, PCC, and the right lOFC between MDD patients and the healthy control within each genotype. **(A)** In the C-homozygous group, cortical thickness in the lOFC of patients with MDD exhibited a significantly greater than that in the healthy controls. **(B)** While in the T-carrier group, patients showed significantly thinner cortical thickness in the left cACC, ISTC, mOFC, and PCC than healthy subjects. (The green bar, the CC genotype of the MDD group; the blue bar, the CC genotype of the HC group; the gray bar, T-carrier of the MDD group; the yellow bar, HC group T-carrier). MDD, major depressive disorder; HC, healthy controls; L, left hemisphere; R, right hemisphere; cACC, caudal anterior cingulate cortex; ISTC, isthmuscingulate; mOFC, the medial orbitofrontal cortex; PCC, posterior cingulate cortex; lOFC, the lateral orbitofrontal cortex. Data are means ± standard error.

**Figure 2:**
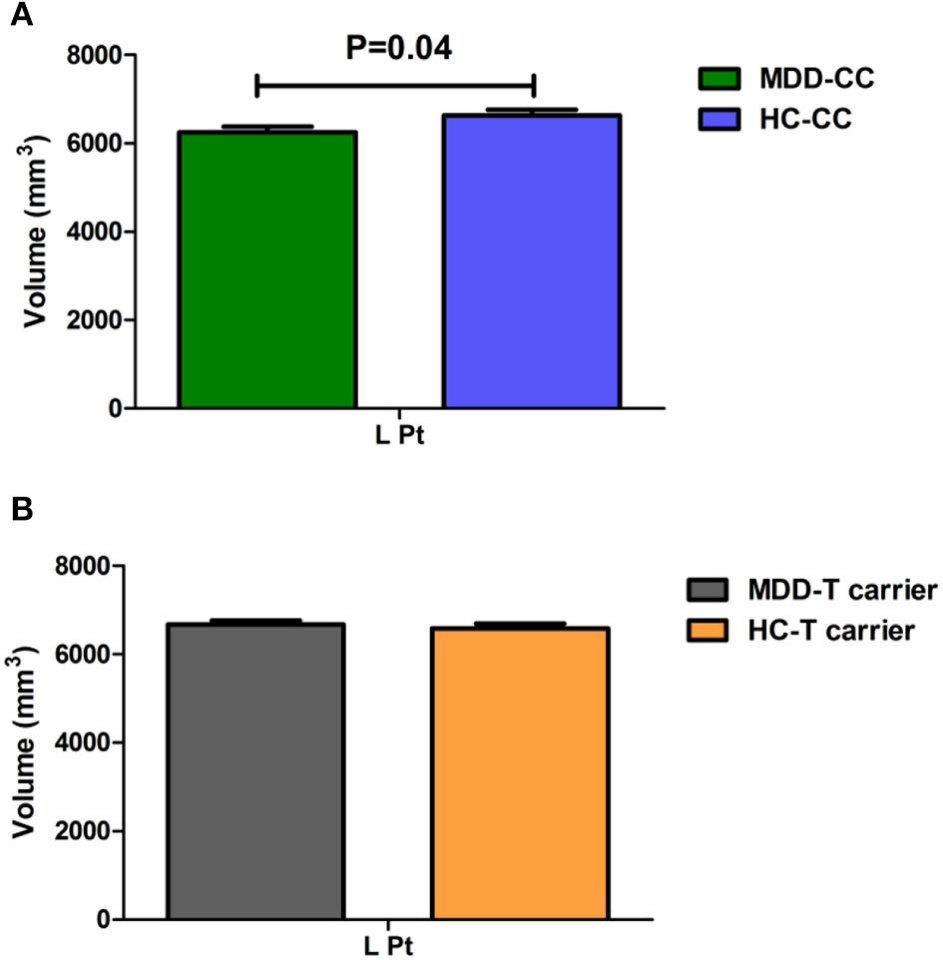
Comparison of the volume value of the left putamen (Pt) between MDD patients and the healthy control within each genotype. **(A)** In the C-homozygous group, MDD patients showed a significantly decreased volume value in the Pt (*P* = 0.040) than healthy controls. **(B)** While In the T allele carrier group, volume value did not differ between patients with MDD and healthy controls. (The green bar, the CC genotype of the MDD group; the blue bar, the CC genotype of the HC group; the gray bar, T-carrier of the MDD group; the yellow bar, HC group T-carrier.). MDD, major depressive disorder; HC, healthy controls; L, left hemisphere; R, right hemisphere; Pt, putamen. Data are means ± standard error.

Moreover, we found a significant trend in interaction effect on the volume of the left putamen (Pt, *F* = 3.872, *P* = 0.05). We used the brain regions with statistically significant diagnosis-by-genotype interactions as ROIs for *post-hoc* analysis. These brain regions included the left cACC, ISTC, mOFC, PCC, and Pt and the right lOFC. We compared CT or volume values extracted from ROIs between MDD patients and HC within each genotype group using a one-way ANCOVA; covariates included those used in the main analysis. In the C-homozygous group, patients appeared to have significantly higher CT values in the right lOFC (*F* = 15.127, *P* < 0.001) and smaller volume values in the left Pt (*F* = 4.386, *P* = 0.040) compared with the HC group. However, there was no significant difference between the T carrier within MDD and HC groups in the above brain regions (*P* > 0.05). In the T allele carrier group, CT values of the left cACC (*F* = 8.356, *P* = 0.004), ISTC (*F* = 4.444, *P* = 0.037), mOFC (*F* = 5.968, *P* = 0.016), and PCC (*F* = 4.031, *P* = 0.047) in depressed patients were significantly thinner than those in the HC group. Likewise, we could not find any significant difference between the C-homozygous group with MDD and HC groups in the above four regions (*P* > 0.05). The detailed data are described in Table 5. The post-analysis of CT in ROIs is shown in Figure 1, and the volume values is displayed in Figure 2.

**Table 5:** *Post-hoc* analysis of the ROIs according to the interaction of “diagnosis × *MTHFR* C677T genotype.”

**Region**	**TT+CT**				**CC**			
	**MDD**	**HC**	**Mean Difference**	**F**	**MDD**	**HC**	**Mean Difference**	**F**
**Cortical thickness (mm)**								
L cACC	2.399 ± 0.036	2.572 ± 0.044	−0.173	8.356**	2.632 ± 0.053	2.539 ± 0.052	0.093	1.409
L ISTC	2.475 ± 0.020	2.545 ± 0.024	−0.070	4.444*	2.563 ± 0.033	2.479 ± 0.033	0.084	2.903
L mOFC	2.341 ± 0.021	2.425 ± 0.025	−0.084	5.968*	2.396 ± 0.024	2.345 ± 0.024	0.052	2.054
L PCC	2.496 ± 0.017	2.555 ± 0.021	−0.059	4.031*	2.556 ± 0.030	2.505 ± 0.030	0.051	1.285
R lOFC	2.574 ± 0.018	2.578 ± 0.023	−0.004	0.015	2.621 ± 0.021	2.499 ± 0.021	0.122	15.127***^a^
**Volume of subcortical gray matter (mm**^**3**^**)**								
L Pt	6.676E3 ± 0.089E3	6.588E3 ± 0.110E3	−0.170E3	0.343	6.247E3 ± 0.126E3	6.639E3 ± 0.125E3	−0.391E3	4.386*

*MDD, major depressive disorder; HC, healthy control; T-carrier, The TT genotype or CT genotype of MTHFR C677T polymorphism; C-homozygous, CC genotype of MTHFR C677T; L, left hemisphere; R, right hemisphere; cACC, caudal anterior cingulate cortex; ISTC, isthmuscingulate; mOFC, the medial orbitofrontal cortex; PCC, posterior cingulate cortex; lOFC, the lateral orbitofrontal cortex; Pt, putamen*.

a *Data are means ± standard error (values of cortical thickness and volume of subcortical). The F and P-values were obtained using one-way analysis of covariance adjusted for age, gender, years of education, and eTIV as covariates as covariates*.

P < 0.05*, P < 0.01**, P < 0.001***.

### Correlation of CT or Subcortical Volume With Illness Duration, Depression, and Anxiety Severity

We investigated correlations between the course of the disease, HAMD score, HAMA score, and CT/volume values of ROIs in MDD patients using partial correlation analysis separately. Consequently, we could not find any significant correlations (all *P* > 0.5). The data are described in detail in Table 6.

**Table 6:** Analysis the correlation between cortical thickness, volume of subcortical values with clinical status of disease.

**Region**	**Illness duration (months)**		**HAMD**		**HAMA**	
	***r-value***	***P-value***	***r-value***	***P-value***	***r-value***	***P-value***
**Cortical thickness (mm)**						
L cACC	−0.012	0.893	0.112	0.210	0.142	0.111
L ISTC	0.010	0.912	0.106	0.235	0.114	0.203
L mOFC	0.000	0.996	−0.049	0.581	−0.076	0.394
L PCC	0.034	0.704	−0.020	0.826	−0.059	0.511
R lOFC	0.128	0.151	0.127	0.155	0.105	0.241
**Volume of subcortical gray matter (mm**^**3**^**)**						
L Pt	−0.048	0.589	−0.101	0.257	−0.149	0.094

*L, left hemisphere; R, right hemisphere; cACC, caudal anterior cingulate cortex; ISTC, isthmuscingulate; mOFC, the medial orbitofrontal cortex; PCC, posterior cingulate cortex; lOFC, the lateral orbitofrontal cortex; Pt, putamen*.

*The r and P-values were obtained using partial correlation analysis, controlling age, gender, years of education, eTIV, and MTHFR C677T genotype as covariates*.

## Discussion

This study firstly investigated the role of *MTHFR* gene polymorphism C677T in pathophysiological processes of MDD in the Han Chinese population in southern China. We observed a significant reduction in the volume of the left NAC in first-episode MDD. Jan Wacker et al. (39) applied multiple methods of the rest-state fMRI, task fMRI, and volume-based measurement to investigate the association between anhedonia, NAC, and rACC. Their findings suggested that anhedonia was associated with reduced reward stimuli in NAC during task fMRI, decreased volume in NAC, and increased rest-state fMRI activity in cACC, which are involved in positive experience-related brain areas. Our research supports structural abnormality of the NAC-prefrontal cortex (PFC) in depression.

Meanwhile, we also found that *MTHFR* C677T allele carriers significantly decreased CT in the left cACC, rACC, frontal pole, right cACC, and supramarginal gyrus compared with the C-homozygous group. There were some studies showing that the C677T allele was associated with gray matter density reduction in the PFC (21), frontoparietal lobe cortex, parieto-occipital lobe cortex, orbitofrontal cortex (OFC), middle frontal gyrus, cingulum, and inferior parietal lobule; volume deficit of the parahippocampus and putamen; or increased volume of the putamen (26, 27, 40, 41), similar to our results. Based on a similar cytoarchitecture and common circuitry and functions, rACC has an integrated effect on the ventral ACC responsible for emotions and the dorsal ACC responsible for cognition function. Therefore, combined with the pathophysiological changes related to *MTHFR* C677T, the *MTHFR* T allele may cause a corresponding physiopathological change by reducing the *MTHFR* enzyme activity, which is related to the reduction of rACC gray matter and the reduction of CT and then participates in the emotional-cognitive changes of depression. Moreover, our research found for the first time that the C677T allele carriers exhibited increased CT in the left cuneus and bilateral pericalcarine. As stated by van Eijndhoven et al. (41), if the critical area (such as OFC) was damaged, other related brain regions [mainly the parts of the default mode network (DMN)] will be compensated by activation or expansion. We also found correlations between C677T risk allele carriers and increased CT of the cuneus and pericalcarine. This may be interpreted as a compensatory increase.

Furthermore, our study verified that there were significant interactions between *MTHFR* C677T and a diagnosis of MDD on CT of the left cACC, ISTC, mOFC, PCC, and the right lOFC. Further, T allele carrier patients yielded reduced CT values in the left cACC, ISTC, mOFC, and PCC compared with T allele carrier HCs. In contrast with the *MTHFR* C-homozygous HCs, the C-homozygous patients demonstrated an increased CT value in the right lOFC. However, in terms of subcortical volume, only the left putamen volume tended to be smaller in C-homozygous MDD patients than in the C-homozygous control group. In short, we could hypothesize that the *MTHFR* C677T allele is a risk factor for the thinning of CT of MDD patients in the left cACC, ISTC, mOFC, and PCC; the C677T homozygote is a protective factor for increased lOFC CT and also allows the volume of the putamen to manifest a decreasing trend in depression. These changed brain regions are included in the structural covariance networks (SCNs), which are defined as having both structural and functional connectivity and modulated by *MTHFR* C677T. Our explanation for these results is that abnormalities in the CT or subcortical volume accompanied by genetic risk conferred by *MTHFR* C677T might be related to the disturbances of neural circuitry involved in emotion processing, which could contribute to a depressive mood. The histopathological alterations within and between structures in DMN and the limbic–cortical–striatal–pallidal–thalamic (LCSPT) circuits may promote dysregulation in emotional behavior and other cognitive aspects of depressive syndromes (40). Growing evidence suggest that *MTHFR* enzyme deficiency caused by *MTHFR* C677T is frequently accompanied by changes in methyl patterns and metabolic disturbance, resulting in methylation of choline and hampered monoamine neurotransmitters (42). Defective *MTHFR* may associate with a series of central nervous system (CNS) cell molecular changes, which negatively affect neuroplasticity in adults.

In sum, these results supported previous literatures and our hypotheses and indicated that *MTHFR* C677T may be involved in one of the pathological mechanisms of depression by affecting the brain structure of LCSPT, especially the thickness of the cACC. In addition, a recent meta-analysis concluded that folate may hold value as an adjuvant treatment for individuals with depression (43). As far as we know, this is the first research investigating the relationship between genetic variants of *MTHFR* C677T and the cortex–subcortical structural changes in patients with MDD.

There are some limitations in the present study. First, our sample is still limited. Second, this study did not measure plasma Hcy, folate, SAM, methyl, etc. at the beginning of the study. Third, we did not find significant differences in HAMD and HAMA scores between *MTHFR* T carriers and C-homozygous groups. Fourth, important environmental factors related to depression, such as childhood trauma, were not included in the study design. In the future, it is necessary to improve the research design to confirm the results.

## Data Availability Statement

This article contains previously unpublished data and datasets are available on request to the corresponding author.

## Ethics Statement

The studies involving human participants were reviewed and approved by the Institutional Review Board of Kunming Medical University. The patients/participants provided their written informed consent to participate in this study.

## Author Contributions

ZL and YC: substantial contributions to conception and design, acquisition of data, or analysis and interpretation of data. BH, JX, LP, CZ, and ZS: drafting the article or revising it critically for important intellectual content. XX, YC, and ND: final approval of the version to be published. All authors contributed to the article and approved the submitted version.

## Conflict of Interest

The authors declare that the research was conducted in the absence of any commercial or financial relationships that could be construed as a potential conflict of interest.
